# Can a Mate‐Finding Allee Effect Be Elicited to Help Eradicate Invasive Mammals?

**DOI:** 10.1002/ece3.72711

**Published:** 2025-12-19

**Authors:** Dean P. Anderson, Sam Gillingham, M. Cecilia Latham, A. David M. Latham

**Affiliations:** ^1^ New Zealand Institute for Bioeconomy Science Lincoln New Zealand; ^2^ Independent Contractor Brisbane Queensland Australia

**Keywords:** demographic stochasticity, mate finding, *Mustela erminea*, pheromones, population control, recruitment, simulation, stoats, survivorship

## Abstract

The Allee effect is an important ecological process that has implications for extinction of endangered species and for assisting the management of invasive species. Few instances of Allee effects have been quantified in nature due to the adaptive resilience of species and the difficulty in empirically linking ecological mechanisms to population change. While Allee effects could theoretically assist with invasive species eradications, the mechanism by which a management intervention could achieve both component and demographic Allee effects has not been tested. We developed an individual‐based simulation model to explore whether the intentional disruption of animal behaviour could elicit a mate‐finding Allee effect to facilitate the eradication of an invasive mammal. We used stoats ( *Mustela erminea* ) as a test species and generalised the results by examining the sensitivity of Allee effects to a range of biological attributes and management scenarios. The ecological challenge was to turn the colonising adaptations (high mobility, olfactory communication and delayed placental implantation) into vulnerabilities for Allee effects. Following an initial population reduction through trapping, reproductive pheromone decoys were deployed to disrupt mate finding. When key ecological, management and technological conditions were met during the deployment of decoys, an increase in the probability of eradication demonstrated a demographic Allee effect. The mate‐finding Allee threshold occurred only at very low densities, indicating the importance of population control. The Allee effect increased with increasing number of deployed decoys and attractiveness to divert movement direction away from potential mates. Increasing the bias in random walk behaviour towards nearby decoys reduced mate finding, whereas low‐biased random walk induced by detection of multiple scents increased reproduction. Increasing survival rates increased longevity and the time for individuals to find mates, which decreased the probability of an Allee effect. The modest increased probability of eradication under optimal biological and management conditions demonstrated the resilience of species to mate‐finding Allee effects.

## Graphical abstract

In this paper we report on results from an individual‐based simulation model developed to identify the conditions under which a mate‐finding Allee effect could be elicited to facilitate the eradication of an invasive mammal in conjunction with lethal trapping. The modest increased probability of eradication under optimal biological and management conditions demonstrated the resilience of species to mate‐finding Allee effects.

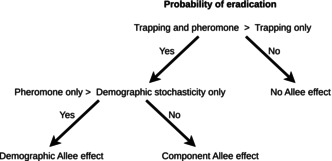

## 1 Introduction

Mate finding is a critical spatiotemporal process in which individuals must locate each other in space during a limited, sexually receptive period. At low population densities, reduced encounter rates may lead to decreased population growth, a phenomenon known as the Allee effect. The Allee effect (Allee 1938; Allee et al. 1949; Berryman 2003) refers to the positive relationship between any component of an individual's fitness and the density of conspecifics (Stephens et al. 1999). When a component Allee effect (e.g., mate finding or predation avoidance) at low population density is stronger than negative density dependence from competition, a demographic Allee effect should decrease the population to local extirpation (Courchamp et al. 1999; Stephens et al. 1999).

Although, Allee effects can put endangered species at risk of extinction (Courchamp et al. 2008), they could assist in the eradication of problematic invasive alien species (Liebhold and Bascompte 2003; Liebhold et al. 2016; Tobin et al. 2009, 2011). If the density of a species can be pushed below its Allee threshold (Berec et al. 2007; Liebhold and Tobin 2008), it could proceed to extinction without further intervention (Caughley and Sinclair 1994). Eradication requires that every potentially reproductive individual either dies or is removed from the area of interest (Bomford and O'Brien 1995). This difficult task is made more challenging by highly fecund species that show high rates of neophobia (i.e., unwilling to interact with baits or traps; Anderson et al. 2016; Garvey et al. 2020; Vattiato et al. 2021; Zub et al. 2022; Johnstone et al. 2024). The elicitation of Allee effects so that the last remaining neophobic individuals would succumb to natural processes could make eradication feasible in combination with standard removal methods.

An example of eliciting an Allee effect is the deployment of artificial pheromone decoys to disrupt mate finding for the last remaining individuals, as behaviour can be altered by experimental odours in the environment (Norbury et al. 2021; Price and Banks 2012; Price et al. 2020). Seeking out decoys would decrease the probability of individuals finding mates during a short reproductive window. A strong response to decoys could result in a demographic Allee effect and increase the probability of eradication. However, eradication could be facilitated by a component Allee effect alone, in the absence of a demographic effect. That is, by reducing reproduction, the population would be constrained to low density until individuals are either removed by standard methods or perish via demographic stochasticity (Lande 1987; Fagan et al. 2002; Petrovskii et al. 2002).

Mate finding results from animal movements, and encounter rates are expected to decrease as population density declines. However, it remains unclear how low density must become before encounter rates are sufficiently reduced to generate an Allee effect, or whether management intervention can further impede mate finding to induce such effects. We developed a spatially explicit, individual‐based simulation model to explore how the adaptive strengths of an invasive, pioneering mammal could be turned into spatial and temporal vulnerabilities in mate finding. We used stoats ( *Mustela erminea* ) as a test species and generalised the results by examining the sensitivity of Allee effects to a range of biological attributes and management scenarios. This provides a mechanistic test of whether behavioural disruption can intentionally elicit a component, and potentially demographic, Allee effect.

Stoats are a problematic invasive predator in New Zealand (Dowding and Murphy 2001; King and Veale 2021; O'Donnell 1996; Whitehead et al. 2008). They exhibit high rates of neophobia (Anderson et al. 2016; Garvey et al. 2020) and their reproductive biology makes them difficult to eradicate. Through the process of delayed placental implantation, females carry within them the potential beginnings of a viable population for most of the year (King and Powell 2007). Kits become sexually mature at approximately 2–3 weeks (Ternovsky 1983), at which time they are usually mated. Adult females are also mated shortly after giving birth. The resulting blastocysts in both juvenile and adults do not implant but float freely in the uterus for 8–11 months. Therefore, a dispersing female can be isolated from a male and still initiate a new population by giving birth to up to 13 kits (King 1994; King et al. 2003; Powell and King 1997). While this is a strength for a pioneering species, it presents a temporal vulnerability if mating can be prevented for one 4‐month mating season (McDonald and Larivière 2001). The time to the next mating season plus the subsequent obligatory period of delayed implantation would result in nearly 2 years without reproduction, which is well into the short life span of a stoat (King and Powell 2007; Erlinge 1983). These females would have a high probability of dying before reproducing.

The mate finding capacity of stoats is enhanced by their mobility (Murphy and Dowding 1994, 1995), swimming ability (King et al. 2014), and use of scent to mark their territories for intraspecific communication (Cloe et al. 2004; Erlinge 1977; Erlinge et al. 1982). Further, males are attracted to odours from oestrous females (Murphy et al. 2022). While managers cannot reduce the distances that animals will travel to find mates, there may be potential to exploit their reliance on olfactory cues. Specifically, this adaptive strength could be turned into a spatial vulnerability by diverting their movements away from potential mates.

Our objective was to identify the conditions under which a mate‐finding Allee effect could be elicited to facilitate the eradication of an invasive mammal in conjunction with lethal trapping. In our simulations, we employed pheromone decoys during the breeding season to disrupt males finding females and non‐pregnant females finding males. The artificial environmental intervention aims to create an Allee threshold (Liebhold and Tobin 2008; Liebhold et al. 2016; Tobin et al. 2011) and trapping pushes the population density below the threshold. We predicted that the presence of pheromone decoys will result in fewer encounters between potential mates in a population that is maintained at low density through trapping. This will result in lower recruitment, potentially driving the population to extinction. This method does not directly remove neophobic individuals from the population, but rather it indirectly removes them as they naturally die and are not replaced through recruitment.

To examine the sensitivity of an invasive species to Allee effects, we used the simulation model to address the following two questions. First, how was the probability of successful eradication influenced by factors related to the effectiveness of the decoys to distract mate‐searching, decoy‐deployment intensity, and the biological parameters of survival and movement? Second, using conditions identified in question 1 that were favourable to achieving eradication, did the deployment of pheromone decoys elicit only a component Allee effect, both a component and demographic Allee effect, or neither? Results reveal the relative vulnerability or resilience of species to mate‐finding Allee effects.

## 2 Methods

### 2.1 Study System

To place our experiment in a realistic setting, we used Resolution Island (45°41.4′ S, 166°41.5′ E), the largest of the near‐shore islands in Fiordland, South Island, New Zealand, as the simulated study area. We did not simulate the habitats or topography of the island per se, but rather the size of the island (208 km^2^), its shape, and the network of traps that already exists on the island (which was partly constrained by topography).

Efforts to eradicate stoats from Resolution Island started in 2008 (Clayton et al. 2011), with the aim of creating the largest island sanctuary in New Zealand for threatened bird species such as the kākāpō ( *Strigops habroptilus* ) and those with large home range requirements such as kiwi (*Apteryx* spp.) and kōkako (*Callaeas* spp.) (McMurtrie et al. 2008). A stoat population currently persists on the Island. While immigration is an ongoing risk for this island, we assume a closed population in order to focus on population control and Allee effects.

### 2.2 Model Description

We developed a spatial individual‐based model of stoats moving in a two‐dimensional landscape. We modelled movement by stoats at two spatiotemporal scales—the period of mate‐searching behaviour (September–January), which does not constrain movement to home ranges, and movement within home ranges outside of the mating season. The trapping network across the landscape was active three times per year (see Section 2.6 below). We assumed that any stoats that encountered and interacted with a trap were killed and removed from the population. We distributed pheromone decoys across the landscape during the oestrous period to disrupt mate finding. Survival, recruitment and dispersal were all modelled, as the simulations were run over multiple years (see Data Availability Statement for Python programming code, data, and movies demonstrating individual movement and population dynamics).

We initialized the model using the parameter ‘initial*N*’, which determined the number of individual stoats remaining on Resolution Island when pheromone decoy management was started. It was a random uniform variate between 6 and 12, which corresponded to the low population number that could be achieved through trapping (Anderson et al. 2016; see Table 1 for parameter ranges). At least one of the initial*N* was a female. From this variable, we used the following two‐step process to determine the number of males and females:

(1) $$\text{initial}N∼\text{uniform}\left(6,12\right)$$

(2) $$n\text{Females}∼\text{binomial}\left(\text{initial}N,0.5\right)$$

(3) nMales=initialN−nFemales

**TABLE 1:** Range of parameter values used in the sensitivity analysis of the model simulating invasive mammal mate‐finding. The model also simulates trapping and the presence of pheromone decoys aimed at exploiting Allee effects to drive a low‐density population to local eradication.

Variable	Description	Range of values
initial*N*	Initial number of individuals	6–12
decoy spacing	Grid resolution of pheromone decoys	300–1000
Number of deployments	Number of pheromone deployments per oestrous period	1 or 2
α	Distance decay home‐range movement	0.01–0.1
δ	Distance decay mate‐searching movement	0.001–0.01
γ	Temporal decay of pheromone decoys	0.005–0.05
habituationDays	Duration of non‐attractiveness towards mate or decoy after an encounter	8–20
*P*(daily survival)	Daily probability of survival	0.9978–0.9986^a^

^a^ Corresponding to an annual probability of survival ranging between 0.45 and 0.61.

**FIGURE A1:**
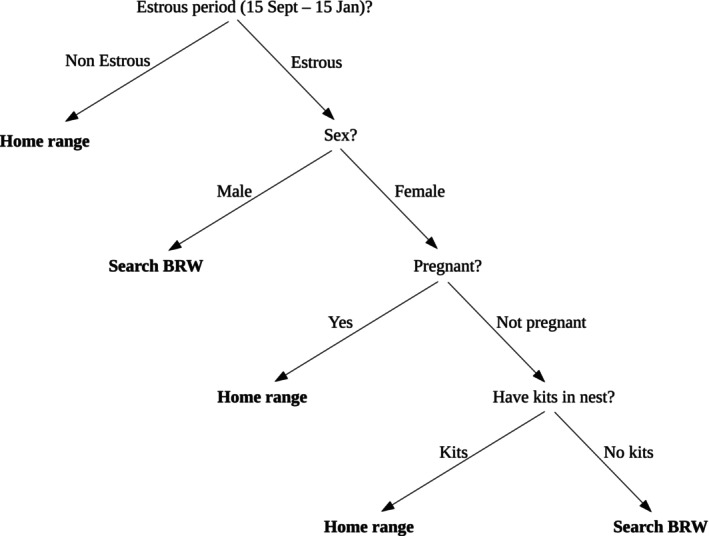
Decision tree for determining stoat movement mode: Home range or biased random walk (search BRW). Estrous refers to the period of the year and not the reproductive state. Pregnant refers to a female having fertilised eggs, but not necessarily implanted placentas.

Depending on the time of the year and sex, individual stoats were in one of three movement states (Figure A1): (1) non‐oestrous period, when all stoats exhibited home range movement behaviour (Clayton et al. 2011; Smith et al. 2015); (2) males exhibiting mate‐searching movement behaviour during oestrous; and (3) females exhibiting mate‐searching movement behaviour if they were not pregnant at the onset of the oestrous period. The latter two states were based on the strong drive to reproduce and that individuals would make extreme efforts to find mates (King and Powell 2007). Oestrous females remained in the mate‐searching state until the end of the oestrous period, unless their eggs were fertilised, in which case they resumed home range behaviour. While stoats may mate between September and January in New Zealand (McDonald and Larivière 2001), we defined the oestrous period as 15 September to 15 January. Because stoats are mainly nocturnal, we simulated their movements only during night time, that is, for 8 h every 24 h. Furthermore, the movements of each stoat were modelled as a time series of 30 min steps (i.e., 16 steps per day).

### 2.3 Home Range Movement

**FIGURE A2:**
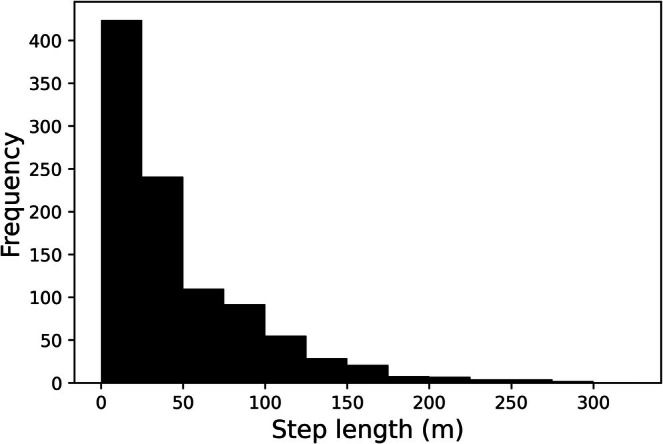
Histogram of step lengths (m) from a Weibull distribution with shape = 1 and scale = 50 m.

**FIGURE A3:**
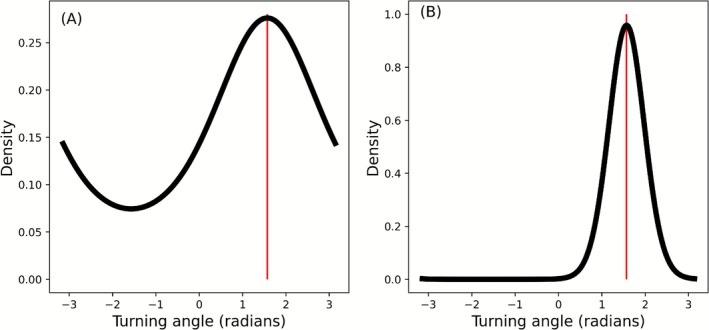
Probability density of turning angles when the centre of attraction (COA) has a bearing of π/2 (vertical red line) and is located at a distance of 700 m. (A) The probability density for home range movement behaviour with α=0.075. (B) The probability density for mate searching movement behaviour with δ=0.005 and 0 days of deployment.

For each movement step of individual *i* at time *t*, the length (Figure A2) and turning angle (Figure A3) were determined by random draws from Weibull and von Mises distributions, respectively (Codling et al. 2010; McClintock et al. 2012). The step length (${\text{stepLength}}_{\mathrm{it}}$) was drawn as follows:

(4) $${\text{stepLength}}_{\mathrm{it}}∼\text{Weibull}\left(\text{shape},\text{scale}\right)$$

where shape = 1 and scale = 50 m for home range behaviour. With shape = 1, the scale represents the average distance moved by a stoat in 30 min.

We used the Von Mises circular distribution to determine the bearing of each movement step in radians within the domain $\left[-\pi ,\pi \right]$. That is, north was 0, and bearings to the west ranged from 0 to −π, and bearings to the east ranged from 0 to π. At each time step t, a random bearing was drawn from a von Mises distribution as follows:

(5) $${\text{bearing}}_{\mathrm{it}}∼\text{vonMises}\left(\mu_{\mathrm{it}},\kappa_{\mathrm{it}}\right)$$

(6) $$\kappa_{\mathrm{it}}=\log\left({\text{dist}}_{\mathrm{it}}^{\alpha }\right)$$

where $\mu_{\mathrm{it}}$ was the bearing to the home range centre of individual *i*, and $\kappa_{\mathrm{it}}$ was a parameter dictating the strength of pull towards the home range centre (i.e., the centre of attraction; COA). $\kappa_{\mathrm{it}}$ was a function of the distance from the current location of stoat *i* to its home range centre (${\text{dist}}_{\mathrm{it}}$) and the distance decay parameter α. As distance from the home range centre increased, the strength of pull increased, and α acted to accentuate or attenuate this effect. Varying this value in the simulation allowed the home ranges to increase (with decreasing $\kappa_{\mathrm{it}}$) or decrease (Table 1). The combination of the shape and scale parameters in Equation (4) and an α of 0.05 would result in home range size of approximately 2.5 ${\mathrm{km}}^{2}$ (Anderson et al. 2016).

### 2.4 Mate‐Searching Movement

**FIGURE A4:**
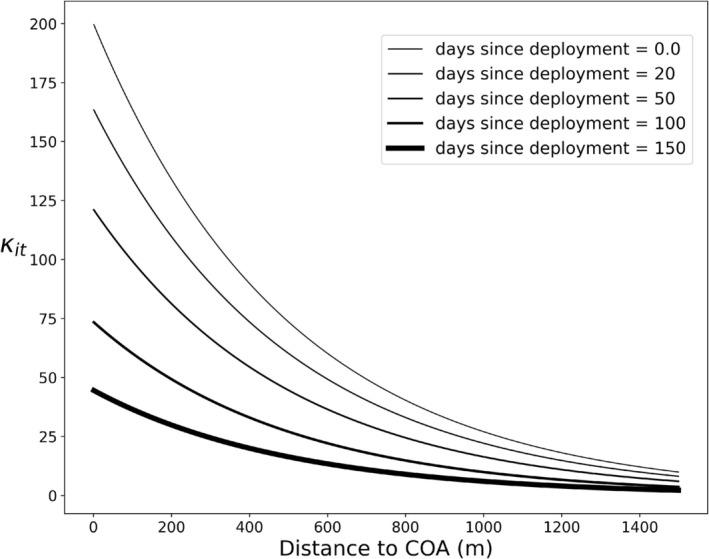
The $\kappa_{\mathrm{it}}$ parameter (i.e., the focus of directional movement), decreases with increasing days since deployment (varying thickness of lines), and distance from the centre of attraction (COA).

**FIGURE A5:**
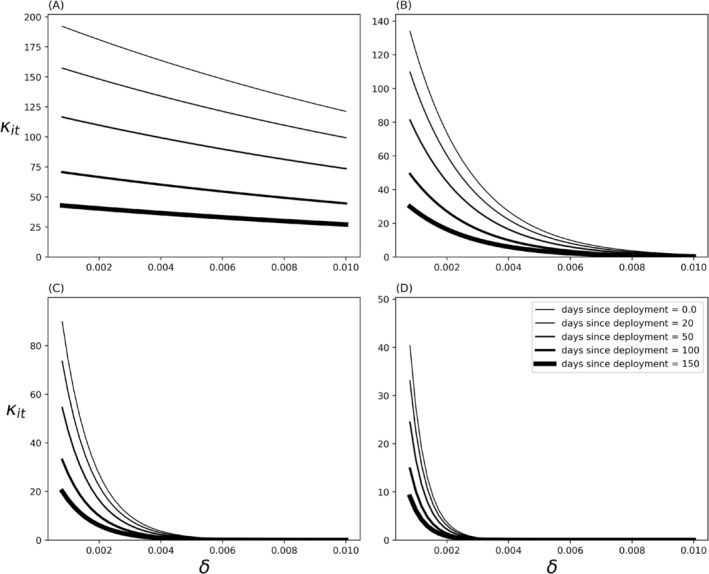
The effect of changing δ on the $\kappa_{\mathrm{it}}$ parameter, that is, the strength of pull towards a centre of attraction (COA), when the COA is located at (A) 50 m, (B) 500 m, (C) 1000 m, or (D) 2000 m from the location of the mate‐seeking stoat. As δ increases, $\kappa_{\mathrm{it}}$ decreases, and this decrease is more acute for larger distances between the mate‐seeking stoat and the COA. This translates into a COA that is very attractive within distances < 50 m, regardless of the value of δ, but that the effect of the COA is negligible for distances > 2000 m for most δ values.

**FIGURE A6:**
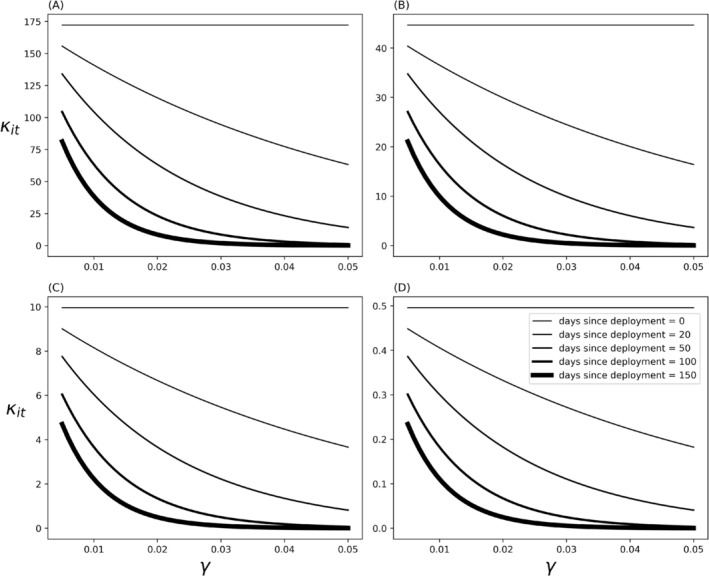
The effect of changing γ on the $\kappa_{\mathrm{it}}$ parameter, that is, the strength of pull towards a centre of attraction (COA), when it is located at (A) 50 m, (B) 500 m, (C) 1000 m, or (D) 2000 m from the location of the mate‐seeking stoat. $\kappa_{\mathrm{it}}$ decreases with increasing γ values, as well as with higher number of days since pheromone deployment and at larger distances between mate‐seeking stoats and the COA.

Mate‐searching behaviour was adopted by female and male stoats to increase the probability of encountering a mate during the oestrous period (15 September–15 January; McDonald and Larivière 2001). If a female was pregnant on 15 September, she was not in oestrus, would maintain home range movement behaviour (Figure A3A), and was not attracted or attractive to males. For stoats in a mate‐searching state, the COA was no longer its home range centre but a selected mate‐searching individual of the opposite sex or a pheromone decoy, if these were available (Figure A3B). The selection of a COA among multiple choices is described below. When far away from the selected COA, the step lengths were drawn from a Weibull distribution with scale = 50 m, as described under the home range movement behaviour (Equation 4). The $\mu_{\mathrm{it}}$ parameter of the von Mises distribution (Equation 5) was the bearing towards the COA, and the $\kappa_{\mathrm{it}}$ parameter was adjusted to be a function of the distance to the COA (${\text{dist}}_{\mathrm{it}}$) and the number of days since decoy deployment (days), if the COA was a pheromone decoy (Figure A4). It was expected that the attractiveness of a decoy would decrease with time (Garvey et al. 2017). The $\kappa_{\mathrm{it}}$ parameter for individuals in a mate‐searching state was calculated as follows:

(7) $$\kappa_{\mathrm{it}}=\left(\frac{1}{\min K}\right)e^{-\delta {\text{dist}}_{\mathrm{it}}}e^{-\gamma \text{days}}$$

where min*K* is the minimum κ value, δ is a parameter dictating how attraction to the COA decays with distance (Figure A5), and γ is a parameter determining how attraction to the COA decays with time since deployment (only for decoys; Figure A6). Empirical estimates of δ and γ do not exist, therefore we used a wide range of potential values (Table 1) that resulted in varying degrees of a correlated random walk (Turchin 1998).

To model the convergence of a stoat on a COA (either a mate or a decoy), we allowed the Weibull scale parameter to be reduced as a stoat approaches its COA. We did this by setting the scale parameter to half the distance between stoat *i* and its COA when the distance was less than the initial scale value (50 m).

A stoat selects a COA by comparing the force of attraction from all potential centres of attraction within a 2.5‐km detection radius. If no stoats of the opposite sex or pheromone decoys are within the detection radius, the ${\text{bearing}}_{\mathrm{it}}$ is the result of a random draw from a uniform distribution ranging between $\left[-\pi ,\pi \right]$. If there are multiple COAs within the detection radius, the force of attraction is determined by the κ of the potential COAs (Equation 7). Mate‐searching stoats have a value of zero for ‘days since deployment’, as their attractiveness does not decline with time, whereas pheromone decoys loose attractiveness with time. For each mate‐searching stoat at each time step, the COAs have to be re‐assessed for their κ values. At time step *t*, the selection of ${\mathrm{COA}}_{i}$ for stoat *i* is done with a random draw from a multinomial distribution:

(8) $${\mathrm{COA}}_{\mathrm{it}}∼\text{multinomial}\left(1,P\left(\mathrm{sel}\right)\right)$$

where *P*(sel) is a vector of multinomial probabilities of selection for the COAs within the detection radius. We calculate *P*(sel) for COA *c* by stoat *i* as

(9) $$P{\left(\mathrm{sel}\right)}_{c}=\frac{\kappa_{c}}{\sum_{c=1}^{C}\kappa_{c}}$$

where $\kappa_{c}$ is the κ value for COA *c* among a total of *C* potential COAs.

At each time step during the oestrous period, an encounter occurs between stoat *i* and the selected ${\mathrm{COA}}_{i}$ if they are within a distance of 25 m. If there are multiple COAs that are encountered, stoat *i* will select to interact with a stoat of the opposite sex over a decoy. If an encounter occurs with a pheromone decoy, stoat *i* attraction to that decoy is disrupted, so that it will not be attracted to that decoy for a number of days defined by the parameter habituationDays. This is based on research showing that animals habituate to and avoid unrewarding odours in the environment (Latham et al. 2019; Price et al. 2020; Norbury et al. 2021). Further, a male that encounters a female will not be attracted to that female for the same number of habituationDays. However, the male will continue to be attracted to other females and decoys.

### 2.5 Mating

If an encounter occurs between a male and an oestrous female, the female is mated and can become pregnant with a 0.9 probability. If the female becomes pregnant, she resumes home range behaviour and does not seek new mates. However, she remains receptive to mate‐searching males, and multiple males can mate with her and sire kits in her next litter. For the duration of the oestrous period, males will still be attracted to her and she can act as a COA for them.

In the wild, female stoats are pregnant for 8–11 months (including an obligate period of delayed implantation), with young being born the following September–October (King and Veale 2021). In our model, if a female becomes pregnant, she remains in that state until the following 30 October, at which time she will give birth. Even if she becomes pregnant at the start of the oestrous period (mid‐September), she has to undergo delayed implantation and give birth the following year (King and Powell 2007). The number of kits (*n*Kits) that are born from each pregnant female is determined from a random draw from a Poisson distribution as follows:

(10) $$\text{nKits}∼\text{Poisson}\left(\text{meanRecruits}\right)$$

(11) $$n\text{FemaleKits}∼\text{binomial}\left(n\text{Kits},0.5\right)$$

(12) nMaleKits=nKits−nFemaleKits

We set meanRecruits = 9, which is the mean number of embryos reported for stoats in New Zealand (King and Moody 1982).

Ten days after the birth, the female will come into oestrus. However, she will maintain home range movement behaviour because she will have kits to care for. The adult female and the female kits are available for mating until 15 January.

Female stoats are sexually mature as early as 2–3 weeks after birth, while they are still blind, deaf and hairless (Ternovsky 1983). Males do not become sexually mature until they are approximately 1 year old. In our model, male and female offspring disperse on 16 January and can set up a territory anywhere on the island. Distances within Resolution Island are well within the dispersing capacity of stoats (Murphy and Dowding 1995). We did not simulate movement behaviour of the offspring between birth and dispersal. For simplicity, we assumed that they stayed at the nest and the adult female provides for them and that is why she displays home range movement behaviour. However, the female offspring in the nest can act as a single COA for males during the oestrous period. If the adult female gets caught in a trap (see below) before the offspring disperse, she and all her offspring die and are removed from the population.

If the female offspring in the nest are mated before 15 January, they become pregnant based on a random draw from a Bernoulli trial with a probability of 0.9. However, they will not give birth until the following 30 October, assuming they survive. The number of male and female kits from female offspring is determined as described in Equations ((10), (11), (12)).

### 2.6 Trapping

We simulated the deployment of the existing Resolution Island trapping network, which was comprised of 2353 DOC150 traps distributed across the island (Anderson et al. 2016). The traps were active during three trapping sessions per year, starting around 20 January, 20 July and 20 November, when new bait was put in each trap. The bait remained attractive for 14 days, after which the traps were no longer active. In our simulations, a stoat encountered a trap if one was within 15 m of its location at the end of each 30‐min movement step. The stoat would interact with an encountered trap and get captured with a probability of 0.2. If the stoat was captured, it was removed from the population.

### 2.7 Natural Mortality

At the end of each day, if a stoat was not captured by a trap, daily survival was determined with a random draw from a Bernoulli trial with $P\left(\text{dailysurvival}\right)$. Daily survival probability was derived from the annual probability of survival, $P\left(\text{annualsurvival}\right)$ (King and Powell 2007), which was a parameter that varied across simulations:

(13) $$P\left(\text{daily survival}\right)=P{\left(\text{annual survival}\right)}^{\left(1/365\right)}$$

### 2.8 Pheromone Decoys

Pheromone decoys were deployed at fixed points within a grid of resolution ranging from 300 to 1000 m. For each iteration of the model, the decoy spacing and the number of decoy deployments (either once or twice during the oestrous period) were chosen at random. Pheromone decoys were always deployed on 15 September, but if there was a second deployment, this occurred on 15 November, or half‐way through the oestrous period. The attractiveness of the pheromone decoys decayed with time, and this was determined by the γ parameter in Equation (7).

### 2.9 Sensitivity Test

The model simulated stoat movements, mating and trapping over 3 years, but if at any point the population decreased to zero individuals, the process stopped. At the beginning of each iteration of the model, a random draw from a uniform distribution was performed for each the following eight input parameters: initial*N*, decoy spacing, number of decoy deployments, α, γ, δ, habituationDays, and *P*(daily survival) (see Table 1 for parameter ranges). We ran 5000 numerical iterations of our model with the objective of obtaining a good coverage of potential combinations of the input parameter values (Dietze 2017; Prowse et al. 2016; García‐Díaz et al. 2021). At the end of each iteration of the model, the response variable was whether or not stoat eradication occurred.

To assess the sensitivity of the probability of eradication success to the model parameters, we regressed the binary eradication outcome across all iterations against the values of the eight random parameters (above) used in the numerical simulations (García‐Díaz et al. 2021). We modelled the eradication event (either a 0 or a 1) in iteration *s*, $E_{s}$, as a Bernoulli process with probability $p_{s}$:

(14) $$E_{s}∼\text{Bernoulli}\left(p_{s}\right)$$

(15) $$\text{logit}\left(p_{s}\right)=\beta_{0}+\sum_{z=1}^{\;8}\beta_{z}\;X_{\mathrm{sz}}$$

where $\beta_{0}$ was the intercept of the logit‐link and $\beta_{z}$ were the coefficients for the eight covariates ($X_{\mathrm{sz}}$). The covariate values were standardised by subtracting their mean and dividing by their standard deviation.

We used a multi‐model approach (Burnham and Anderson 2002) to identify which combination of covariates were most important in determining stoat eradication success. We fitted the data to a null model (no covariates) and five alternative models that included different combinations of covariates (Table 2). Before constructing models, we assessed the level of correlation between pairs of covariates. All pair‐wise comparisons had *r* < 0.5. We estimated parameters for the six statistical models using a Bayesian Markov Chain Monte Carlo (MCMC) regularisation approach (Gelman et al. 2013; Hooten and Hobbs 2015). We assessed the support for each model by comparing their DIC (deviance information criterion) value (Spiegelhalter et al. 2002). We used relatively uninformative priors, $\beta ∼\text{Normal}\left(0,10\right)$, for the intercept and all the slopes of the logistic regression. Convergence for all fitted models was achieved following a burn‐in of 5000 samples. We obtained posterior summaries from 3000 samples after a thinning rate of 20. The MCMC algorithm was written in Python programming language.

**TABLE 2:** Results from five competing models, with associated k (number of model parameters), DIC and ΔDIC values, used to assess the influence of density and attractiveness of pheromone decoys and of biological parameters on the probability of stoat eradication from Resolution Island, New Zealand. Lower DIC values are better, indicating that model 1 provided the best fit to the data.

Model	Variables	*k*	DIC	ΔDIC
1	initial*N*, decoy spacing, number of deployments, α, δ, γ, habituationDays, *P*(daily survival)	9	5942	0
2	initial*N*, decoy spacing, number of deployments, habituationDays	5	6126	185
3	α, δ, γ	4	6273	331
4	decoy spacing, number of deployments	3	6318	376
5	initial*N*, *P*(daily survival)	3	5995	53
6	null model	1	6320	378

**FIGURE 1:**
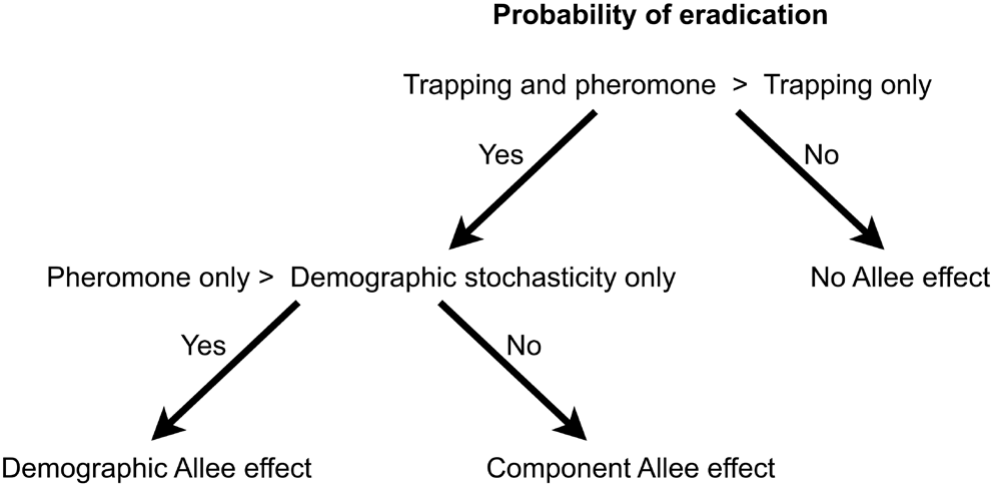
Logic tree for making inference on the post hoc models to determine the whether the deployment of pheromone decoys elicited only a component Allee effect, a demographic Allee effect or neither. If the probability of eradication with the deployment of both trapping and pheromone decoys is greater than with only trapping, then at least a component Allee effect is present. If eradication is more likely with only pheromone deployment (and no trapping) than with neither pheromone or trapping (i.e., demographic stochasticity only), then a demographic Allee effect is present.

The finding of statistically important parameters related to the deployment of pheromone decoys would be indicative of Allee effects. We explored potential Allee effects further by using results from the Bayesian statistical model to design scenarios to test whether the deployment of pheromone decoys elicited only a component Allee effect, both a component and demographic Allee effect, or neither. The first scenario was an experimental‐control to quantify the probability of eradication due only to demographic stochasticity. This scenario assumed that trapping had decreased the population to a low number of survivors (initial*N*), and then no further intervention occurred (no trapping or decoys). The second scenario included the deployment of only traps and no pheromone decoys to quantify the probability of eradication due to trapping and demographic stochasticity. The third scenario involved the deployment of both trapping and pheromone decoys. A higher probability of eradication in this scenario than the trapping‐only scenario would indicate either a component or demographic Allee effect (Figure 1). In the fourth scenario, only pheromone decoys were deployed and there was no trapping. A higher probability of eradication in the pheromone‐only scenario than in the demographic‐stochasticity‐only scenario (i.e., no trapping or decoys) would indicate a demographic Allee effect. For each scenario, we ran 400 independent realisations of the model and calculated the resulting probability of eradication as the number of realisations in which the population was reduced to zero divided by the 400 realisations.

## 3 Results

Results of our simulation modelling showed that management manipulation of the environment has the potential to create an Allee threshold for a highly adapted colonising invasive mammal. In this case study, the demographic Allee effect increased with increasing numbers of deployed decoys and attractiveness to nearby decoys to divert movement direction away from potential mates.

**FIGURE A7:**
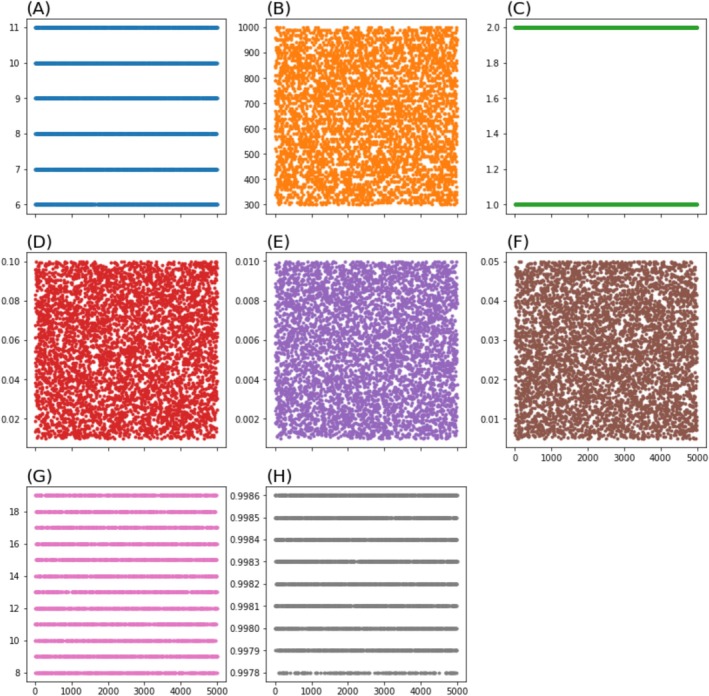
The sample of input parameter values that were randomly drawn to simulate stoat mate‐finding dynamics in the presence of pheromone decoys aimed at enhancing Allee effects: (A) initial*N*, (B) decoy spacing, (C) number of decoy deployments, (D) α, (E) δ, (F) γ, (G) habituationDays, (H) *P*(daily survival). The *y*‐axis is the parameter value and the *x*‐axis is the iteration number (1–5000).

**FIGURE A8:**
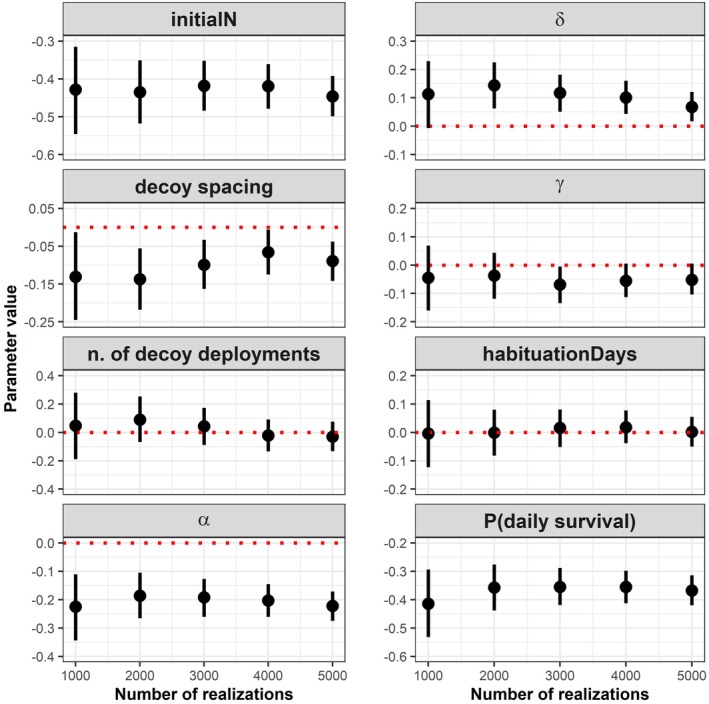
Estimated parameter values (and 95% CI) for each of the explanatory covariates included in our logistic regression model explaining variation in the probability of stoat eradication from Resolution Island using pheromone pollution and trapping. Parameter values were estimated using an increasing number of model realisations, but final parameter estimates were based on results from 5000 realisations.

From the 5000 iterations of the stoat movement and mate‐finding model, we obtained a comprehensive sample of the parameter space for the eight covariates of interest (Figure A7). Furthermore, 5000 model iterations ensured that β estimates for each covariate had reached a stable level (Figure A8).

The model that resulted in the lowest DIC included all of the covariates for which we simulated parameter values (Model 1 in Table 2). The next best model (Model 5) included only two biological parameters; however, this model had ΔDIC of 53 relative to model 1, suggesting that there was significantly less support for it. All fitted models had ΔDIC < 2 relative to the null model.

Model 1 included three biological parameters (initial*N*, habituationDays, *P*(daily survival)), two parameters describing the way pheromone decoys were deployed (decoy spacing, number of decoy deployments), and three movement parameters (α, δ, γ). Of the covariates included in model 1, initial*N* and *P*(daily survival) had the largest influence on probability of stoat eradication from Resolution Island (Table 3). The probability of eradication decreased if there was a larger initial population of stoats and with increasing daily probability of survival. The probability of stoat eradication increased with increasing resolution of decoy deployment (i.e., smaller spacing between decoys).

**TABLE 3:** Posterior parameter estimates and associated 95% credible intervals (CI) for the best statistical model (Table 2) describing the influence of density and attractiveness of pheromone decoys and of biological parameters on the probability of stoat eradication from Resolution Island, New Zealand. The covariate values were standardised by subtracting their mean and dividing by their standard deviation before including them in analyses.

Parameter	Mean	Low CI	High CI
Intercept	−0.7720	−0.8450	−0.6990
initial*N*	−0.4450	−0.4990	−0.3920
decoy spacing	−0.0900	−0.1400	−0.0380
number of deployments	−0.0250	−0.1270	0.0790
α	−0.2220	−0.2740	−0.1700
δ	0.0670	0.0160	0.1180
γ	−0.0520	−0.1050	0.0050
habituationDays	0.0030	−0.0490	0.0540
*P*(daily survival)	−0.3680	−0.4200	−0.3160

Two movement parameters, α and δ, had an important influence on the probability of stoat eradication. The probability of stoat eradication decreased with increasing α values, suggesting that when attraction towards the home range centre is strong during home range movement (Equation 6), individuals will stay closer to it and be less exposed to traps. Conversely, probability of stoat eradication increased with increasing δ values. As δ increases, stoats will be less distracted by weak scents from distant decoys or potential mates, and will focus in on more proximal COAs (Figure A3). The less stoats get distracted by far away pheromone scents, the more they will hone in on nearby pheromone decoys. High distraction from distant decoys (i.e., small δ) will result in a random walk, which should increase the probability of encountering a mate‐searching stoat of the opposite sex.

Credible intervals for the γ temporal decay in pheromone attractiveness and habituationDays parameters spanned zero. This indicates that they had an uncertain influence on the probability of stoat eradication.

The important effect of parameters related to the pheromone decoy deployment (decoy spacing and δ) provided evidence that a mate finding Allee effect was operating in this simulated system. Based on the results from Model 1 (Table 3), we used the following parameter values in our post hoc models to determine whether a component or demographic Allee effect was elicited: intialN=6; number of deployments =2; decoy spacing =300; α=0.02; δ=0.01; γ=0.0051; habituationDays=19; *P*(daily survival) =0.9981. The scenario with both traps and pheromone decoys had a higher probability of eradication than with trapping only; 0.64 and 0.57, respectively. This indicates the presence of an Allee effect (Figure 1). The pheromone‐only scenario had a 0.05 probability of eradication, which was slightly higher than the 0.025 for the no‐intervention (i.e., demographic‐stochasticity scenario). These findings suggest that modest component and demographic Allee effects were elicited in this system under favourable conditions (Figure 1).

## 4 Discussion

Results from our exploratory simulation experiment showed that important management, biological and technological conditions must be met to elicit a demographic Allee effect to drive an invasive mammal to local extinction. The component Allee effect in this study was the disruption of mate finding using pheromone decoys (Stephens et al. 1999). However, even under optimal conditions (i.e., favourable parameter values from the statistical model), only a weak Allee effect was achieved. We were able to confirm the presence of an Allee effect by the measured increase in the probability of eradication with pheromone decoy deployment in addition to trapping (0.57 to 0.64) and demographic stochasticity (0.025 to 0.05). The marginal increase illustrates the difficulty in eliciting an Allee effect in a highly adapted pioneering species (Gascoigne et al. 2009) that otherwise would not be vulnerable. Nevertheless, the novel strategy offers a potential passive means to facilitate the removal of the last survivors, which are the most difficult and costly to dispatch (Cruz et al. 2009). Even when an Allee effect is theoretically possible and conditions are favourable for it to occur, its realisation is not deterministic and will entail significant stochasticity. An observed Allee effect, like all events, is probabilistic.

The initial population size (initial*N*) was the most influential parameter in the best fitting model predicting eradication success, as indicated by the standardised coefficients (Tables 2 and 3). For example, the probability of achieving eradication increased 2.5‐fold when pheromone deployment started after trapping had reduced the population size to 2.8 stoats/100 km^2^, relative to starting deployments at a larger population of 5.3 stoats/100 km^2^. The former density is predicted to be below the threshold (Liebhold and Tobin 2008) so that Allee effects could be exploited for an eradication strategy.

The decoy spacing was another management parameter that exerted an important influence on the probability of eradication by eliciting a mate‐finding Allee effect. Our results show that this component Allee effect was strong enough to cause a demographic Allee effect that could lead to the extirpation, but only if trapping first reduced the population to low numbers.

The most important biological attribute that influenced the probability of eradication was daily survival rate (*P*(daily survival)). Increased longevity would buffer a population from extinction by increasing the number of years available to find a mate and reproduce. Even within the limited annual survival range of 0.45 to 0.61 for stoats, this factor was very important in the best fitting model (Table 3). We only explored 3 years of combined trapping and pheromone decoy deployment. If management scenarios ran for more years, it is possible that the negative effect of daily survival rate on the probability of eradication would persist but be somewhat reduced. That is, eradication may still be achievable with relatively high rates of *P*(daily survival), but it could take more years of trap and decoy deployment, requiring increased financial investment.

Home‐range movement behaviour is another biological attribute that influenced the probability of eradication. The negative relationship between the home‐range movement parameter (α) and the probability of eradication demonstrated the importance of coordinated and well‐designed deployment of traps. Because home‐range size decreases with increasing α (due to attraction to the home‐range centre; Equation 6), the layout of traps must be adapted to the expected home‐range size of the target species (Anderson et al. 2022; Mackenzie et al. 2022; Smith et al. 2015). Trap spacing must decrease with decreasing home‐range size. We did not explore alternative trapping strategies, because our aim was to explore how pheromone decoys could be used to complement an existing and realistic trapping regime.

Our results show the way that a mate‐searching individual detects and reacts to distant pheromone scents is also important in eliciting an Allee effect, as indicated by the positive relationship between the pheromone distance parameter (δ) and the probability of eradication (Table 3). With high δ values, animals will home in on nearby pheromone decoys and be less distracted by far away pheromone scents. This will result in stoats going from decoy to decoy in a biased‐random walk (Turchin 1998), and will ignore potential mates that are further away than the decoys (i.e., mate‐finding disruption). In contrast, with low δ values, the pool of potentially attractive COAs (mates or decoys) increases, and the probability of selection across the COAs becomes more equal (see multinomial process in Equations 8 and 9). This movement pattern will be more similar to a random walk and should increase the probability of encountering a mate‐searching individual of the opposite sex, increasing the probability of mating and reducing the probability of eradication.

Another biological process that we simulated was the period of non‐attractiveness towards a decoy or an individual of the opposite sex after an initial interaction. However, this had little influence on the probability of eradication as evidenced from the 95% credible intervals for this parameter (habituationDays) overlapping zero. The lack of effect could be due to the biased random walk induced by the mate‐searching behaviour (Erlinge 1977; Erlinge and Sandell 1986; Sandell 1986) and pheromone decoys, which would reduce the tendency to return to previously encountered mates or decoys.

The pheromone distance decay parameter (δ) reflects both the technological development/identification of suitable pheromone compounds and the biological reaction to decoys releasing those compounds which causes a change in movement behaviour. Success of any effort to elicit an Allee effect via disruption of mate finding will depend on the development of effective pheromone decoys. Evidence suggests that there is potential to disrupt mate‐finding during the oestrous period. Mustelids are olfactory‐sensitive, as they use odours to mark their territories for intraspecific communication (Berzins and Helder 2008; Cloe et al. 2004; Erlinge 1977; Erlinge et al. 1982), males are attractive to odours from oestrous females (Murphy et al. 2022), and artificially deployed odours have been shown to alter their behaviour (Garvey et al. 2017). Further, artificial odours have been successfully deployed in situ to alter the behaviour of invasive predators, including mustelids, and improve survival of threatened birds (Norbury et al. 2021; Price and Banks 2012).

While free‐ranging animals will respond to natural and artificially deployed olfactory cues in the environment (Erlinge 1977; Garvey et al. 2017; Latham et al. 2019; Murphy et al. 2022; Norbury et al. 2021; Price and Banks 2012), their reaction is expected to decline with time as compounds decay (Bytheway et al. 2013). The effectiveness of disrupting mate finding is expected to increase with decreasing rates of decay in the attractiveness of the pheromone compounds. Our results showed that the probability of eradication increased with decreasing γ, the temporal decay rate parameter of pheromone decoys (Table 3, Equation 7). The γ posterior was clearly negative, but the 95% credible intervals slightly overlapped zero, suggesting a marginally important relationship. This could be due to a compensatory effect of redeployment of fresh decoys (number of deployments) and the concurrent trapping, which could remove individuals not distracted by decoys. While our results also showed that 95% credible intervals of the number of deployments parameter overlapped zero, the problem of decaying decoy attractiveness should be overcome by repeated deployments and concurrent trapping.

The idea for this study emerged from the challenge posed by the last few individuals remaining after eradication attempts across a range of species, which have proven very difficult to remove as they appear to be shy or neophobic (Anderson et al. 2016; Cruz et al. 2009; Garvey et al. 2020; King and Moody 1982; Zub et al. 2022). Given that female stoats are effectively pregnant all the time (King and Powell 2007) and a single litter can seed a viable population, it only takes the survival of a single female to prevent successful eradication. Their high mobility (King and Veale 2021; Murphy and Dowding 1994, 1995) combined with their reproductive biology makes them a well‐adapted colonising species and naturally resilient to Allee effects. The applied ecological challenge of this study was to exploit the spatial and temporal vulnerabilities to mate‐finding Allee effects, such as olfactory communication, short life span, limited oestrous period, and the prolonged period between fertilisation and birth due to delayed placental implantation (King and Powell 2007). While the elicited Allee effect was modest, the eradication of problematic invasive species will likely benefit from small incremental contributions of multiple tools and techniques.

## Author Contributions

**Dean P. Anderson:** conceptualization (lead), data curation (lead), formal analysis (equal), funding acquisition (lead), investigation (lead), methodology (lead), project administration (lead), software (lead), supervision (equal), validation (lead), visualization (equal), writing – original draft (lead), writing – review and editing (lead). **Sam Gillingham:** conceptualization (supporting), formal analysis (supporting), methodology (supporting), software (equal), writing – original draft (supporting), writing – review and editing (supporting). **M. Cecilia Latham:** data curation (equal), formal analysis (equal), methodology (equal), software (equal), visualization (equal), writing – original draft (equal), writing – review and editing (equal). **A. David M. Latham:** investigation (equal), methodology (equal), writing – original draft (equal), writing – review and editing (equal).

## Funding

This research was funded by Manaaki Whenua Landcare Research Strategic Science Investment Fund.

## Conflicts of Interest

The authors declare no conflicts of interest.

## Data Availability

Python programming code, data, and movies demonstrating individual movement and population dynamics are available at https://doi.org/10.5061/dryad.wstqjq30m.
